# Interactive influences of emotion and extraversion on visual attention

**DOI:** 10.1002/brb3.2387

**Published:** 2021-10-17

**Authors:** Robert C. A. Bendall, Shaunine Begley, Catherine Thompson

**Affiliations:** ^1^ Directorate of Psychology & Sport School of Health and Society University of Salford Salford UK

**Keywords:** change detection, emotion, extraversion, visual attention, visual search

## Abstract

**Introduction:**

Emotion has been shown to influence selective visual attention. However, studies in this field have revealed contradictory findings regarding the nature of this influence. One possible explanation for the variation in findings is that affective inter‐individual differences impact both attention and emotion and may therefore moderate any influence of emotion on attention. The current work is a novel investigation of the effects of induced emotional states and the traits of extraversion and neuroticism on visual attention. This allowed a direct investigation of any impact of extraversion and neuroticism on the way in which emotion influences attention.

**Methods:**

Participants were induced into positive, neutral, and negative emotional states before completing a change detection flicker task in which they were required to locate a change to a real‐world scene as quickly and accurately as possible.

**Results:**

Participants scoring higher in extraversion were more accurate but slower at detecting changes. Importantly, this was particularly evident when induced into a negative emotional state compared to a neutral emotional state. Neuroticism had no impact on attention.

**Conclusions:**

The current study provides evidence that extraversion can moderate the influence of negative emotion upon visual attention and may help to explain some of the contradictory findings in this research area. When considered independently, increased trait levels of extraversion were associated with improved change detection. Individuals higher in extraversion appear better equipped to regulate negative emotion compared to individuals lower in extraversion, supporting research linking extraversion to affective reactivity and models of psychopathology.

## Graphical abstract

The ability of affective traits to influence the effect of emotion on change detection is currently unknown. Extraversion independently improved change detection and interacted with emotion to guide the allocation of attention. Individuals higher in extraversion appear better equipped to regulate negative emotion compared to individuals lower in extraversion supporting research linking extraversion to affective reactivity and models of psychopathology.

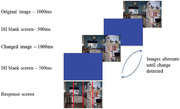

## 1 INTRODUCTION

The visual world is cluttered, and it is impossible to attend to all items and areas simultaneously. Priority is therefore given to the most relevant areas or objects. This "biasing" of attentional resources, known as selective visual attention, is subject to a range of influences and is dependent upon top‐down and bottom‐up processing (Schneider & Shiffrin, 1977). Top‐down processing is characterized by goal‐directed behavior (e.g., directed attention to a target during visual search), whereas bottom‐up processing refers to the automatic capture of attention by salient information in the environment regardless of task demand (Itti & Koch, 2000).

Selective attention (together with other aspects of executive function) is underpinned by the recruitment of the prefrontal cortex (PFC), in particular the dorsolateral prefrontal cortex (dlPFC) (Curtis & D'Esposito, 2003; Miller & Cohen, 2001). The dlPFC shares parallel anatomical connections with the posterior parietal cortex (Katsuki & Constantinidis, 2012), a region of the parietal lobe associated with a number of cognitive processes including selective attention (Behrmann et al., 2004). For instance, it has been revealed that dorsal attention networks, including the intraparietal cortex and superior frontal cortex, are involved in preparing and conducting goal‐directed selection of stimuli and responses (Corbetta & Shulman, 2002). Further, the PFC has been shown to play a crucial role in the switching of top‐down attention allocation (Rossi et al., 2009).

In addition to being crucial to selective attention, research has demonstrated that the PFC is involved in emotional processing during cognitive tasks (for reviews see Dolcos et al., 2011; 2014). It is suggested that emotional processing comprises neural activation resulting from interactions between the *hot* emotional system found in limbic regions (e.g., the amygdala) and *cold*, higher order emotional systems located in the PFC (Dolcos et al., 2011; Fossati, 2012). Additionally, direct and indirect anatomical connections exist connecting the *hot* and *cold* emotional neural systems (Fossati, 2012; Ray & Zald, 2012). Given that the same top‐down resources are used in emotional processing and the allocation of selective visual attention, it follows that emotion may have an impact on attention.

This suggestion is supported by a wide range of research findings and theoretical models, one of which is the broaden‐and‐build theory (Fredrickson, 2001). The theory proposes that positive emotions, including joy, interest, contentment, pride, and love, have the ability to “broaden” an individual's “thought‐action repertoires.” It is also suggested that positive emotions “build” an individual's “enduring resources,” including physical resources (e.g., life longevity; Danner et al., 2001), intellectual resources (e.g., theory of mind; Leslie, 1987), social resources (e.g., relationship quality; Aron et al., 2000), and psychological resources (e.g., resilience; Fredrickson et al., 2003). Specifically, the theory posits that, over time, experiencing positive emotions will have a cumulative effect; enabling an individual to become more creative, knowledgeable, resilient, socially integrated, and healthy, and providing them with resources that can be utilized as necessary in the future. The theory predicts that positive emotions will therefore have a beneficial impact on visual attention by expanding available resources. Frederickson outlines that negative emotions have the opposite effect, preventing one from thinking broadly and from building lasting psychological reserves.

Empirical research has provided support for the predicted broadening impact of positive emotion on visual attention. In an eye‐tracking study, participants were presented with three images (one located in the center of the screen and two situated in the periphery). Participants induced into a positive mood state made more fixations on the peripheral stimuli compared to those induced into a neutral mood state and the researchers concluded that this reveals a broadening of attention under positive moods (Wadlinger & Isaacowitz, 2006). Using a modified version of the Eriksen flanker task (Eriksen & Eriksen, 1974), Rowe et al. (2007) also provided evidence for a broadening of visuospatial attention in positive moods. They presented participants with a central target that was flanked by distracters and manipulated the distance between the targets and distracters. Findings showed that participants induced into a positive mood suffered more interference from far distance distracters compared to those induced with neutral and sad moods. This suggests that in positive moods attention will expand, allowing an individual to process more information (regardless of whether this is relevant or irrelevant to the task).

Despite the evidence for a broadening effect of positive emotion, there is now a growing body of literature that casts doubt on the broaden‐and‐build theory (Bendall & Thompson, 2015; Bruyneel et al., 2013; Grol & Raedt, 2014; Taylor et al., 2017). Bruyneel et al. (2013) conducted a partial replication of the flanker experiment of Rowe et al. (2007) and found no evidence for a broadening of attention on the basis of positive mood. The researchers did find that overall response times were longer in the negative mood condition (regardless of flanker location or compatibility), although they acknowledged this effect may have been due to a lack of counterbalancing in the experiment. In a study investigating the allocation of spatial attention Bendall and Thompson (2015) induced participants into positive, neutral, and negative emotional states and asked them to locate central and peripheral changes in a change detection flicker task (Rensink et al., 1997). Whilst central changes were detected faster than peripheral changes, emotion had no impact on this, providing no evidence for a broadening effect.

Given the contrasting findings regarding the effects of emotion on selective attention, it is important to consider the factors that may be contributing to these mixed results. Cognitive theories attempt to explain behavior in terms of average group‐level performance, however, these models often fit less accurately when they are applied to individuals (Parasuraman & Giambra, 1991). Therefore, a possible explanation for the differing findings regarding the effects of emotion on attention is differences between individuals. One possible method to explore the influence of individual differences on the relationship between emotion and visual attention is to investigate personality traits. Extraversion and neuroticism are two personality traits that feature in models of psychopathology and are linked to affective reactivity (Clark, 2005; Watson et al., 1994). Extraversion is characterized by optimism, energetic engagement with the world, and enjoyment of social contact (DeNeve & Cooper, 1998; Diener et al., 1992; Eysenck, 1990; John et al., 2008; Watson et al., 1994). Extraversion is also associated with positive emotion and happiness and extraverts report higher wellbeing and more positive experiences than introverts (Costa & McCrae, 1980; DeNeve & Cooper, 1998). Neuroticism has been conceptualized in terms of emotional instability and heightened reactivity to stress and aversive environmental stimuli, and it reflects the tendency to experience negative emotions (John et al., 2008; Watson et al., 1994).

Using a rapid serial visual presentation task MacLean and Arnell (2010) found that higher levels of neuroticism were associated with a larger attentional blink (AB), whilst greater extraversion predicted smaller ABs. Early theories to account for the AB effect suggest that it occurs due to limited processing capacity whereby individuals are unable to process information presented in close temporal proximity (Chun & Potter, 1995; Raymond et al., 1992). These results would therefore indicate that extraverts and individuals low in neuroticism can process more information and consequently suffer from the AB to a lesser extent. Neuroticism and extraversion have also been found to predict attentional performance during change detection. It has been demonstrated that higher accuracy was associated with higher levels of extraversion but lower levels of neuroticism, suggesting that these individuals demonstrate improved performance in demanding tasks that measure the allocation of attention (Hahn et al., 2015). Research, therefore, shows that personality traits associated with emotional reactivity can influence attention and cognitive resources.

Consequently, the current work proposes that personality traits, and specifically neuroticism and extraversion (which are associated with emotional reactivity), may moderate the effects of emotion on attention and this may be one reason for the differing findings in the literature. Higher levels of extraversion and lower levels of neuroticism appear to be associated with increased cognitive resources, allowing for improved performance in tasks measuring attention. If emotion influences attention by influencing attentional capacity, it may be proposed that such personality traits will interact with the effects of emotion. For instance, due to their increased availability of cognitive resources, individuals higher in extraversion and lower in neuroticism may not show the predicted impact of positive emotion in comparison to neutral emotion. Similarly, individuals who are lower in extraversion and higher in neuroticism, and who may have limited attentional resources, will suffer from a negative mood state to a greater extent than those higher in extraversion and lower in neuroticism. This is because negative emotion will reduce the capacity of an already limited set of resources, whereas because those with higher levels of extraversion (for example) have more resources available to them, they will be protected from the impact of negative emotional states.

Research testing these predictions is lacking and this limits the understanding of how emotional processing impacts attention. The current work provides an important contribution towards understanding the interaction between emotion and personality on visual attention and is the first study to adopt a change detection task to investigate such interactions. Given the links between specific traits and emotion, the aim was to investigate whether the influence of emotion on attention is affected by extraversion and/or neuroticism. Participants were induced into positive, negative, and neutral emotion states and asked to detect changes to neutral scenes in a change blindness flicker paradigm (following the method of Bendall & Thompson, 2015). Three conditions of emotional state were used because it has been argued that positive and negative (as well as neutral) conditions need to be included in studies investigating affective processing to allow more precise conclusions to be reached (Bendall et al., 2016; Carretié, 2014). Self‐reported levels of extraversion and neuroticism were recorded. It was predicted that higher levels of neuroticism would result in poorer performance, whereas higher levels of extraversion would be associated with improved change detection. Following the findings from Bendall and Thompson (2015) that emotion does not impact visual attention during a change detection flicker task, it was predicted that, when analyzed in isolation, emotion would have no influence on performance. Crucially, it was hypothesized that emotion would have an influence when considered together with the individual difference traits of extraversion and neuroticism. Specifically, it was predicted that a negative emotional state would impair performance for those reporting lower levels of extraversion compared to those higher in extraversion. It was also predicted that performance under a negative emotional state would be reduced for participants higher in neuroticism but not for those lower in neuroticism. Moreover, it was predicted that a positive emotional state would improve performance for individuals reporting lower levels of extraversion compared to individuals higher in levels of extraversion. It was also predicted that a positive emotional state would improve performance for individuals with higher levels of neuroticism compared to individuals with lower levels of neuroticism.

## 2 METHODS

### 2.1 Participants

A sample size calculation aiming to achieve statistical power of .80 with an alpha criterion of .05 and effect size of .25 suggested that a sample of 28 participants was required. An opportunity sample of 30 students (26 female) from the University of Salford aged between 19 and 30 years (*M* = 23.47, *SD* = 3.45) participated in this experiment. Participants were excluded if they had a diagnosed psychiatric condition. Written informed consent was gained from each participant and ethical approval was obtained from the School of Health Sciences Ethical Approval Committee at the University of Salford. All methods were carried out in accordance with the relevant guidelines and regulations. Where appropriate participants received course credit for participating.

### 2.2 Design

A mixed design was used with three independent variables. A within‐participants variable included *emotion* induced prior to the change detection task (positive, neutral, or negative). Two between‐participant variables consisted of levels of *neuroticism* (higher or lower by median split), and levels of *extraversion* (higher or lower by median split). Since the study did not make predictions regarding possible interaction effects between extraversion and neuroticism, and to maximize the power of the statistical analyses, neuroticism and extraversion were analyzed separately resulting in a 3 × 2 analytical framework. The dependent variables consisted of accuracy (percentage correct) and response time (in seconds) to detect the changes. A measure of positive and negative affect was also recorded to validate the method for inducing emotion.

### 2.3 Materials

The experiment was designed and run using E‐Prime (Psychological Software Tools, Inc.) and participants completed the experiment using a Viglen Intel Quad Core computer with a 60 Hz, 19‐inch monitor. Emotion was manipulated by presenting participants with visual images from the International Affective Picture System (IAPS; Lang et al., 2008) prior to completing the change detection task. The images were selected on the basis of emotional valence and a total of 60 images were used, 20 positive (mean valence 7.65), 20 neutral (mean valence 4.62), and 20 negative (mean valence 2.35; see Table S1). Twenty additional positive images (mean valence 7.87) were presented at the end of the experiment (see Table S2). The purpose of the images was to induce emotion based on valence and therefore the images were only controlled for valence and no other characteristics were considered.

A total of 180 neutral images were used for the change detection task. One central change and one peripheral change were made to 36 original images (both indoor and outdoor scenes) making a further 72 images. All changes were deletions (one item in the scene disappeared) and care was taken to ensure that changes were all a similar size. Central changes were made within the center of each image (within an area measuring 512 × 384 pixels) and peripheral changes were made outside of this area. There were an equal number of peripheral changes made on the right and left sides of the images. For each changed image a response screen was also created. This consisted of the original image separated into four equal sections that each contained a red letter (A, B, C, and D) to allow participants to indicate the location of a change. All images in the change detection task were presented in color and measured 1024 × 768 pixels (subtending 30.13° × 22.82° of visual angle) and were identical to those used by Bendall and Thompson (2015).

The Positive and Negative Affect Schedule (PANAS), a 20‐item self‐report measure, was used to record participant mood after presentation of affective stimuli (Watson et al., 1988). The measure consists of 20 words that describe positive and negative feelings and emotions. The words were presented in a random order and for each word participants were asked to “indicate to what extent you feel this way right now, that is, at the present moment” on a scale of 1 (very slightly or not at all) to 5 (extremely). The PANAS provides a measure of positive affect from the summed rating of all positive words and a measure of negative affect from the summed rating of the negative words. The minimum score for each measure is 10 (indicating low affect) and the maximum is 50.

The Revised NEO Personality Inventory (NEO‐PI‐R; Costa & McCrae, 2008) was administered to measure levels of neuroticism and extraversion. The 240‐item self‐report scale includes questions relating to personality characteristics and traits that are measured on a 5‐point Likert scale from 0 (strongly disagree) to 4 (strongly agree). Each trait is measured by 48 questions, the minimum score for each trait is 0 (indicating low levels) and the maximum is 192. The full inventory was administered; however, the focus of this study was scores for extraversion and neuroticism.

### 2.4 Procedure

**FIGURE 1:**
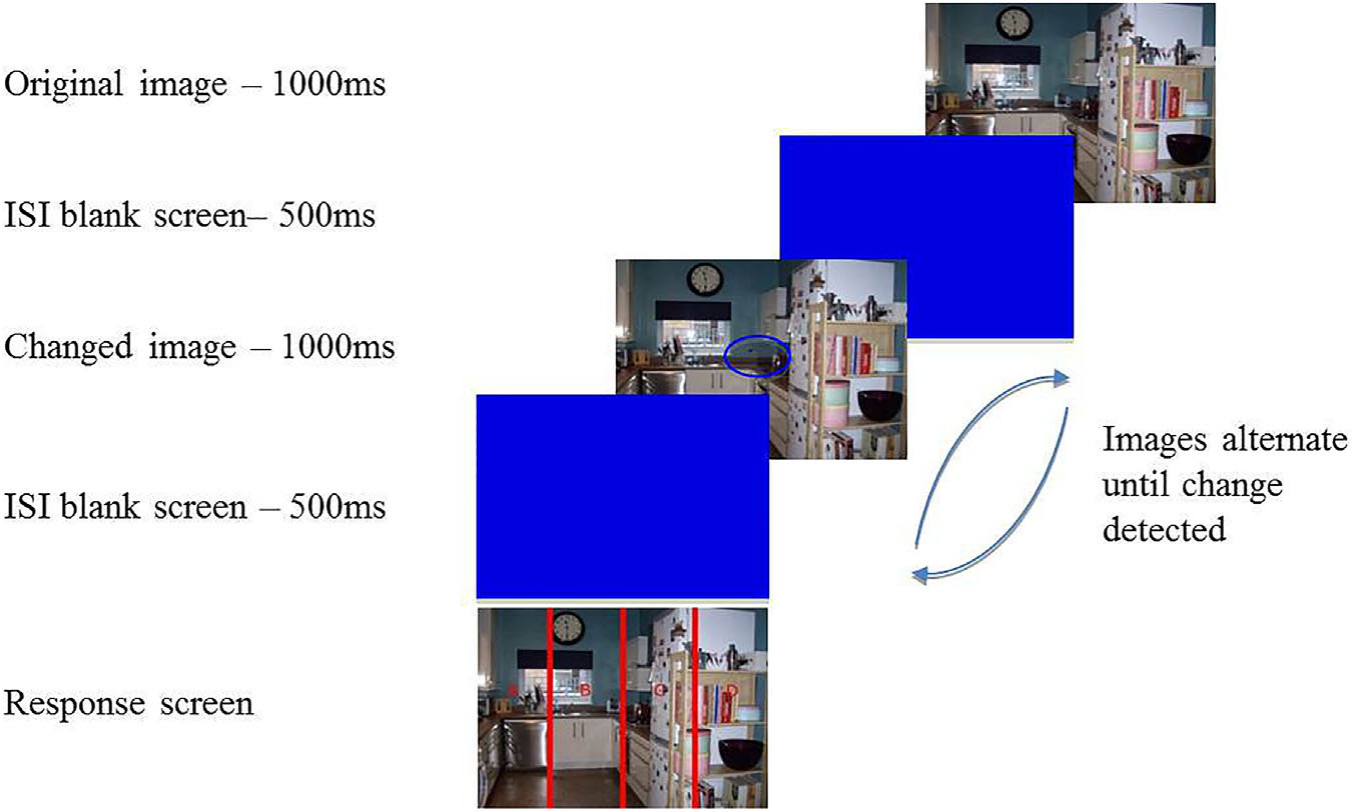
Illustration of the change detection flicker task. Participants were presented with an image for 1000 ms followed by an inter‐stimulus interval (ISI; blank screen) for 500 ms. A changed image was then presented for 1000 ms followed by another ISI for 500 ms. This procedure continued until the participant identified the change in the scene and pressed the spacebar. They were then required to state where the change occurred by pressing the corresponding letter on the response screen. In this example, the change was located in the center of the image and the correct response was "B." Replicated with permission from Bendall and Thompson (2015)

After providing written informed consent participants completed the NEO‐PI‐R (∼16 min) and were verbally given full instructions about the task. Participants were also provided with onscreen instructions throughout the experiment. The experimental procedure followed that used by Bendall and Thompson (2015). Participants were seated approximately 22 inches from the screen and pressed the spacebar when ready to begin. They first viewed 20 images from the IAPS which were either positive, neutral, or negative (e.g., 20 positive images). Each image was presented once. The images were shown for 5000 ms in a random order, with a 500 ms inter‐stimulus interval (ISI; blank white screen) separating each one, and participants were asked to view these pictures naturally (this part of the experimental procedure lasted 110 s). Once all images had been presented participants were instructed to complete the PANAS to validate the IAPS‐based emotion induction procedure (∼60 s). Following this, participants pressed the spacebar to begin the change detection task. In each trial within this task, an image was presented for 1000 ms followed by a blue blank screen for 500 ms. The changed image was then presented for 1000 ms, again followed by the blue screen for 500 ms. Participants were asked to search for the change between the two images and the images continued to alternate until participants pressed the spacebar to indicate they had located the change (Figure 1). A response screen was then presented, and participants reported the location of the change by pressing the relevant key on the keyboard (A, B, C, or D). Participants were told that the changes may be difficult to spot and if they were unable to locate the change they had the option of pressing "9" to end a trial; however, they were asked to only use this as a “last resort.” See Supplementary Figure 1 for an example of trial stimuli. Twenty‐four change detection trials were completed, consisting of 12 central changes and 12 peripheral changes (6 to the left and 6 to the right), and each trial was unique. All trials were presented in random order and this task took approximately 4 min to complete.

Participants completed three experimental blocks that each followed the above procedure (viewing of the IAPS images, completion of the PANAS, the change detection task). In the first block the 20 IAPS images were either positive or negative (counterbalanced across participants). In the second block the 20 images were always neutral, and in the final block the 20 images were again positive or negative depending on the valence of the first set of images. The 20 images used within each block were presented randomly.

**FIGURE 2:**
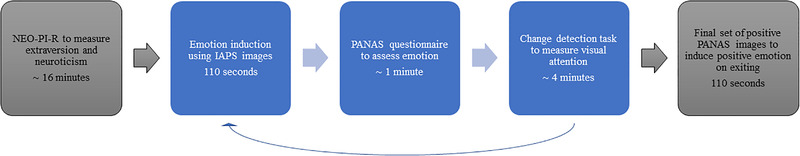
Experimental procedure. After completing the NEO‐PI‐R participants completed three experimental blocks. Each experimental block comprised three elements. A block started with the presentation of 20 images from the IAPS to induce emotion (positive, neutral, or negative). Subsequently, participants completed the PANAS to assess emotion induction success. Following this, participants completed the change detection task. The three experimental blocks were presented pseudorandomly (positive–neutral–negative or negative–neutral–positive). Finally, participants were presented with 20 positive images before leaving the laboratory. On average participants took 38 min to complete the full experimental procedure

Following completion of the final experimental block participants were shown 20 further positive images from the IAPS which were presented for 5000 ms each with an ISI of 500 ms (taking 110 s). This was to make certain they were induced into a positive mood when leaving the laboratory. On average participants took 38 min to complete the full experiment. The experimental procedure is shown in Figure 2.

### 2.5 Statistical analysis

All statistical analyses were performed using IBM SPSS Statistics for Windows, Version 25. A significance level of *p* = .05 was adopted for all main effects and planned contrasts with a more stringent significance threshold of *p* = .0125 used to investigate significant interactions. Where the assumption of sphericity had been violated, degrees of freedom were corrected using Greenhouse–Geisser estimates of sphericity.

To assess the success of the emotion induction procedure, scores from the PANAS were analyzed. The positive affect scores and negative affect scores were analyzed independently using two separate repeated measures ANOVAs. For each analysis, planned comparisons were conducted to compare scores in the neutral emotion induction condition to scores in the negative emotion induction condition and scores in the positive emotion induction condition. Additionally, to compare levels of affective reactivity between extraversion and neuroticism groups, *t*‐tests were used to analyze negative affect scores for individuals higher and lower in extraversion and higher and lower in neuroticism under negative emotion conditions, as well as positive affect scores for individuals higher and lower in extraversion and higher and lower in neuroticism under positive emotion conditions.

Accuracy (percentage correct) and response time (seconds) data were analyzed using mixed measures ANOVAs. To maximize power the influence of extraversion and neuroticism on performance in the change detection tasks were assessed separately using a series of 3 (emotion) × 2 (extraversion or neuroticism) mixed measures ANOVAs. Two ANOVAs analyzed the accuracy data, one for extraversion and one for neuroticism. Two further ANOVAs analyzed the response time data, one for extraversion and one for neuroticism. Each individual difference trait comprised a between‐participants variable with two conditions (higher or lower levels of the trait). Induced emotion was treated as a within‐participants variable (positive, neutral, or negative). Planned comparisons were utilized to compare the neutral emotion condition to the positive and negative emotion conditions. Further interactions were explored using *t*‐tests with an adjusted significance threshold of *p* = .0125 to correct for multiple comparisons.

## 3 RESULTS

Overall change detection accuracy was 84.84% and participants took an average of 9.02 s to correctly identify the change. Outliers were removed at ± 2 standard deviations from the mean resulting in one participant being excluded from the analysis. Average extraversion scores across both groups were: higher extraversion group (*M* = 131.80, *SD* = 16.28), lower extraversion group (*M* = 100.13, *SD* = 6.74). Average neuroticism scores across both groups were: higher neuroticism group (*M* = 121.87, *SD* = 19.30), and lower neuroticism group (*M* = 85.40, *SD* = 8.70).

**FIGURE 3:**
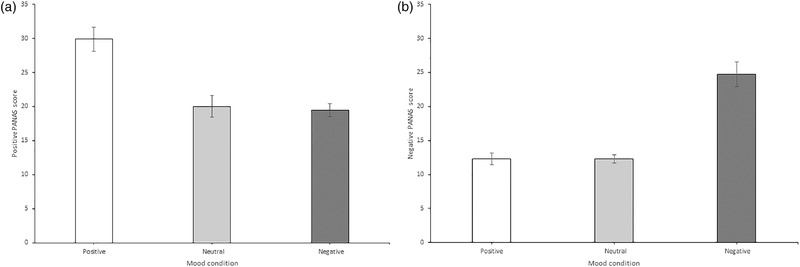
Mean Positive and Negative Affect Schedule (PANAS) scores for each induced mood condition. Self‐reported mood varied across the conditions for both (a) positive affect (b) and negative affect. Error bars = standard error of the mean

The manipulation to induce emotion was successful. Analysis of the positive affect scores showed a significant effect of emotion, *F*(2, 56) = 23.55, *MSE* = 42.28, *p* < .001, *η*_p_^2^ = .46. Planned comparisons show that viewing positive stimuli significantly increased positive mood scores compared to viewing neutral stimuli (*M* = 29.90, *SD* = 9.48 vs *M* = 20.03, *SD* = 8.47), *F*(1, 28) = 29.45, *MSE* = 95.77, *p* < .001, *η*_p_^2 ^= .51; Figure 3. There were no differences in positive affect after viewing negative stimuli (*M* = 19.48, *SD* = 5.26) compared to neutral stimuli, *F*(1, 28) = .16, *MSE* = 55.33, *p* = .693, *η*_p_^2 ^= .01; Figure 3.

For negative affect Mauchly's test indicated that the assumption of sphericity had been violated, therefore degrees of freedom were corrected using Greenhouse–Geisser estimates of sphericity (∑ = .60). Analysis of negative affect also showed a significant effect of emotion, *F*(1.206, 37.781) = 37.95, *MSE* = 65.80, *p* < .001, *η*_p_^2 ^= .58. Planned comparisons revealed that viewing negative stimuli significantly increased negative mood scores compared to viewing neutral stimuli (*M* = 24.76, *SD* = 9.69 vs *M* = 12.28, *SD* = 3.13), *F*(1, 28) = 59.09, *MSE* = 76.47, *p* < .001, *η*_p_^2 ^= .68; Figure 3. There was no difference in negative affect between the positive (*M* = 12.28, *SD* = 4.52) and neutral conditions, *F*(1, 28) = .00, *p* = 1.000, *MSE* = 25.14, *η*_p_^2 ^= .00; Figure 3.

For positive affect in the positive induced emotion condition there was no difference in positive affect scores for individuals higher in extraversion (*M* = 28.93, *SD* = 9.57) and individuals lower in extraversion (*M* = 30.13, *SD* = 9.78), *t*(28) = .340, *p* = .369, *d* = .12. There was also no difference in positive affect scores in the positive emotion condition for individuals higher in neuroticism (*M* = 27.13, *SD* = 9.87) and individuals lower in neuroticism (*M* = 31.93, *SD* = 8.85), *t*(28) = 1.402, *p* = .086, *d* = .51. For negative affect in the negative induced emotion condition individuals higher in extraversion (*M* = 21.20, *SD* = 8.92) showed reduced negative affect scores compared to individuals lower in extraversion (*M* = 27.53, *SD *= 9.82), *t*(28) = 1.850, *p* = .038, *d* = .68. There was no difference in negative affect scores in the negative emotion condition for individuals higher in neuroticism (*M* = 23.73, *SD* = 10.17) and individuals lower in neuroticism (*M* = 25.00, *SD* = 9.66), *t*(28) = .350, *p* = .365, *d* = .13.

When analyzing performance in the change detection task, the effect of emotion was non‐significant for both accuracy, *F*(2, 54) = .66, *MSE* = 151.77, *p* = .521, *η*_p_^2 ^= .24, and reaction time, *F*(2, 54) = 2.00, *MSE* = 8.52, *p* = .145, *η*_p_^2 ^= .07. Neuroticism also had no significant effect on accuracy, *F*(1, 27) = .18, *MSE* = 264.49, *p* = .671, *η*_p_^2 ^= .01, or reaction time, *F*(1, 27) = .75, *MSE* = 11.12, *p* = .393, *η*_p_^2 ^= .03. For accuracy, the interaction between neuroticism and emotion was non‐significant, *F*(2, 54) = .882.76, *MSE* = 162.03, *p* = .421, *η*_p_^2 ^= .03. The interaction between neuroticism and emotion for reaction time was also non‐significant, *F*(2, 54) = .08, *MSE* = 10.44, *p* = .926, *η*_p_^2 ^= .00.

**FIGURE 4:**
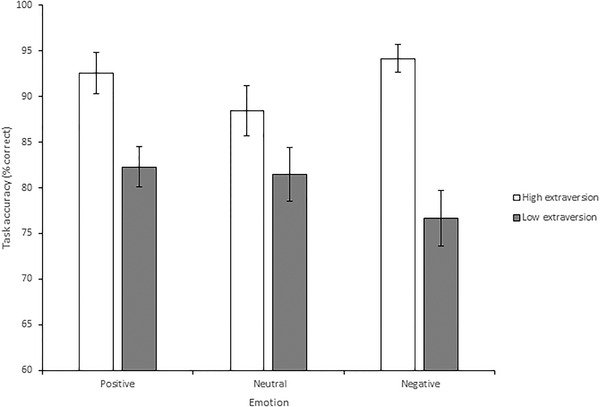
Mean percentage accuracy in the change detection task. Individuals high in extraversion detected changes more accurately than individuals low in extraversion. Accuracy to identify changes during the negative emotion condition was greater for those high in extraversion compared to those low in extraversion, whereas no differences in accuracy were evident during neutral and positive mood conditions on the basis of extraversion. Error bars = standard error of the mean

**FIGURE 5:**
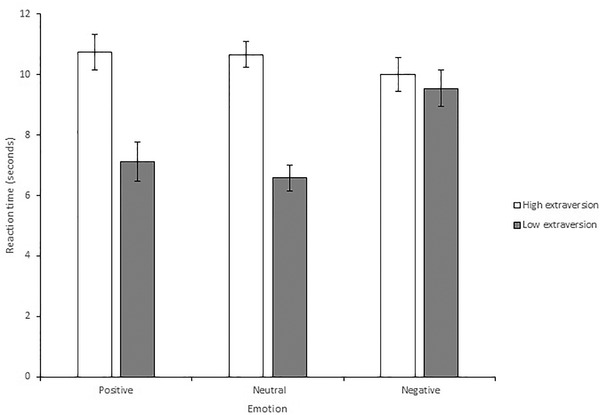
Mean reaction time in the change detection task. Individuals high in extraversion took longer to detect changes than those in the low extraversion group. Response times during the neutral emotion condition were quicker for the low extraversion group compared to the high extraversion group, whereas during the negative and positive emotion condition no differences in response time were evident between the two extraversion groups. Error bars = standard error of the mean

There was a significant effect of extraversion on accuracy, *F*(1, 27) = 10.02, *p* = .004, *MSE* = 194.23, *η*_p_^2 ^= .27; Figure 4. Individuals in the higher extraversion group detected changes more accurately than those in the lower extraversion group (*M* = 91.70%, *SD* = 8.08 vs. *M* = 80.11%, *SD* = 11.25). There was also a significant effect of extraversion on reaction time, *F*(1, 27) = 15.44, *MSE* = 7.27, *p* = .001, *η*_p_^2 ^= .36; Figure 5. Individuals higher in extraversion took longer to detect changes than those in the lower extraversion group (*M* = 10.48s, *SD* = 2.86 vs. *M* = 7.70s, *SD* = 3.32). This suggests a speed‐accuracy trade‐off for participants reporting higher levels of extraversion.

For accuracy the main interaction effect between extraversion and emotion was non‐significant, *F*(2, 54) = 2.76, *MSE* = 151.77, *p* = .072, *η*_p_^2 ^= .09; Figure 4. Planned comparisons do not however require the omnibus F to reach significance so were still completed. Comparisons that compared the positive and negative conditions to the neutral condition showed no significant difference in the extraversion groups between the positive and neutral conditions, *F*(1, 27) = .35, *MSE* = 445.59, *p* = .560, *η*_p_^2^ = .01. However, when comparing the negative and neutral conditions there was an effect of extraversion, *F*(1, 27) = 8.62, *MSE* = 185.83, *p* = .007, *η*_p_^2 ^= .24; Figure 4. The *t*‐tests to further explore this interaction showed that when induced into a negative mood accuracy was higher for individuals higher in extraversion compared to the lower extraversion group, *t*(20.55) = −4.050, *p* = .001, *d* = 1.49. In contrast, when induced into a neutral mood there was no difference in accuracy for individuals higher and lower in extraversion, *t*(27) = −1.334, *p* = .193, *d* = .50.

There was also a significant interaction between extraversion and emotion for reaction time, *F*(2, 54) = 6.18, *MSE* = 8.52, *p* = .004, *η*_p_^2 ^= 0.19. The comparisons showed no interaction between emotion and extraversion in the positive and neutral conditions, *F*(1, 27) = .24, *MSE* = 14.88, *p* = .626, *η*_p_^2^ = .01. However, there was an interaction when comparing the negative condition to the neutral condition, *F*(1, 27) = 12.58, *MSE* = 14.30, *p* = .001, *η*_p_^2 ^= .32; Figure 5. The additional *t*‐tests showed that when induced into a neutral mood reaction time was quicker for individuals lower in extraversion compared to those higher in extraversion, *t*(27) = −5.705, *p* < .001, *d* = 2.13. In contrast, when induced into a negative mood there was no difference in reaction time between individuals higher and lower in extraversion, *t*(27) = ‐.628, *p* = .535, *d* = .23.

## 4 DISCUSSION

The aim of the study was to investigate whether extraversion and neuroticism moderate the effect of emotion on visual attention. Past research has shown conflicting findings regarding the impact of emotion on attention and one possible contributing factor to this is the influence of personality traits. The present study is a first attempt to explore this, and the traits of extraversion and neuroticism were selected as a focus given their relationship to emotional reactivity and to performance in the change detection flicker task (the method used to assess attention). Participants were induced into positive, negative, and neutral mood states and asked to detect changes made to real‐world images as quickly and as accurately as possible. It was predicted that higher levels of neuroticism would have a detrimental impact upon change detection, whereas higher levels of extraversion were predicted to improve change detection performance. It was expected that emotion would have no impact on change detection when considered in isolation (as found by Bendall & Thompson, 2015, using the same experimental paradigm). However, due to the predicted benefit of extraversion during emotional processing, and the detrimental impact of neuroticism on emotional processing, when the effects of extraversion and neuroticism are considered, it was predicted that individuals higher in neuroticism and individuals lower in extraversion would show reduced performance under negative conditions compared to neutral conditions. Additionally, it was predicted that individuals lower in extraversion and individuals higher in neuroticism would show improved performance under positive emotion conditions.

Extraversion was shown to influence accuracy overall in the task whereby individuals higher in extraversion detected changes more accurately than individuals lower in extraversion. This finding supports previous work showing that extraversion is able to predict performance during a change detection task with higher levels of extraversion related to improved accuracy (Hahn et al., 2015). Extraversion was also shown to influence reaction time. Individuals higher in extraversion were slower to detect changes than individuals lower in extraversion. Taken together, these results suggest that in relation to extraversion, participants exhibited a speed‐accuracy trade‐off in performance.

An explanation for the current finding is that extraverts possess additional attentional resources compared to introverts. Indeed, Eysenck (1982) suggests that extraverts have greater resource availability than introverts and this hypothesis is supported by a number of studies adopting a range of experimental paradigms (e.g., the attentional blink, MacLean & Arnell, 2010; for a review see Matthews, 1997). The current study used the change blindness flicker paradigm to investigate visual attention and this is a demanding task. Participants are presented with an image, followed by a blank screen, then the image is presented again but with a change that participants are required to locate and identify. This process repeats until the change is identified (Rensink et al., 1997), and due to the removal of the usual motion artifacts using the blank screen, identification of the change is difficult. Individuals are required to hold information in their minds in order to compare it to the following representation. The finding that those higher in extraversion show greater accuracy in a change detection task suggests that they have more capacity (resources) for creating and storing a representation and so are better able to complete the task.

Research also suggests that the relationship between extraversion and visual attention may be partially dependent upon the difficulty of experimental tasks. For instance, research has shown that introverts perform better than extraverts in low prevalence visual search tasks such as baggage screening and radiology assessments, which are characterized as easy, boring, and repetitive (Peltier & Becker, 2017). Additionally, a recent study adopting a simple visual search task found no association between extraversion and visual attention (Bendall et al., 2021). Moreover, differences in executive function have been observed on the basis of extraversion. Campbell et al. (2011) demonstrate that extraverts perform better than introverts on updating tasks, whilst introverts outperform extraverts on set shifting tasks. The relationship between task difficulty and extraversion has also been investigated using a divided attention test with single and dual task conditions where set size is varied at 3, 4, or 5. Szymura and Nȩcka (1998) demonstrate that in single task conditions no differences in accuracy were evident based on extraversion. However, during dual task conditions, no differences in performance were evident based on extraversion during easier task conditions (set size of 3), whereas during difficult trials (set size of 4 or 5) extraverts perform more accurately than introverts. These studies suggest that task difficulty may influence the impact of extraversion on visual attention. This could be explored in more detail in future work by carefully manipulating the demands of visual attention tasks. Additional work may also include eye movements to study the effects of emotion, extraversion, and neuroticism on visuospatial attention as findings have suggested that eye movements are related to personality traits including neuroticism, extraversion, and curiosity (Hahn et al., 2015; Hoppe et al., 2018; Risko et al., 2012).

Overall, the current result suggests that extraversion is linked to enhanced attentional performance during a change detection flicker task due to increased ability to allocate attention and search the whole scene. The observed increase in reaction time for individuals higher in extraversion may be due to these individuals conducting a more exhaustive (but accurate) visual search, and so it can be suggested that individuals lower in extraversion did not have the required resources to search the whole scene demonstrated by quicker (but less accurate) responses.

As predicted, there was no main effect of emotion on performance, in accordance with past research using the same paradigm (Bendall & Thompson, 2015), however, there was an indication that personality traits may be moderating the influence of emotion on attention. Supporting the hypothesis, extraversion was shown to interact with emotion to impact change detection performance. In the negative condition accuracy to identify changes was higher for individuals higher in extraversion than individuals lower in extraversion, whereas no differences in accuracy between individuals higher and lower in extraversion were evident during the neutral (and positive) emotion conditions. This suggests that when individuals are in negative moods, those who are lower in extraversion have reduced ability to allocate attention. Further, extraversion was also shown to interact with emotion for change detection response time. Response times during the neutral (and positive) emotion condition were quicker for the lower extraversion group compared to the higher extraversion group, whereas no differences in response time were evident between the higher and lower extraversion groups during the negative emotion condition. This interaction effect is the result of a slowing of reaction time under negative emotion conditions for individuals lower in extraversion compared to the same individuals when in positive and neutral emotion conditions. It therefore appears that emotion does impact visual attention, but this may depend on extraversion levels of individuals (potentially accounting for some of the non‐significant effects of emotion in past studies).

One possible mechanism for this result comes from the proposition that emotion regulation requires the recruitment of resources and brain regions also involved during completion of the change detection task, thus reducing the availability of top‐down attentional resources. Emotion regulation is broadly defined as the initiation of a conscious or non‐conscious effort to start, stop, or modulate an emotion (Gross, 2015). Areas of the PFC are implicated in emotion regulation (Buhle et al., 2014; Ochsner et al., 2012) and these same structures are also involved in selective attention (Curtis & D'Esposito, 2003; Miller & Cohen, 2001; Rossi et al., 2009) therefore it may be expected that they would compete for resources. Importantly, however, it may be that individuals higher in extraversion are able to successfully regulate their emotions more effectively than individuals lower in extraversion. Successful regulation means fewer resources are devoted to this process, allowing more resources to be directed to the change detection task. In turn this means that performance will be impaired under negative conditions that require effortful emotion regulation, but only for those lower in extraversion who have fewer resources from the outset. The analysis of negative affect during the negative emotion condition supports this argument as individuals lower in extraversion demonstrated heightened affective reactivity to the negative emotion induction. This finding complements our behavioral findings and interpretation suggesting that the negative emotion condition impacted individuals lower in extraversion to a greater extent than individuals higher in extraversion. Additional evidence to support the proposed link between negative emotion, extraversion, and emotion regulation comes from studies showing (1) that high levels of extraversion are linked to improved emotion regulation as well as negatively correlated with emotion dysregulation (Kokkonen & Pulkkinen, 2001), (2) that poor emotion regulation is related to increased negative affect as well as the development of affective disorders such as depression (Aldao et al., 2010; Brockman et al., 2017; Latif et al., 2019; Suri & Gross, 2015), and (3) that affective disorders are linked to low levels of extraversion (American Psychiatric Association, 2013; Shankman & Klein, 2003). We argue that individuals higher in extraversion are better able to regulate negative emotion thus affording increased levels of cognitive resources compared to individuals lower in extraversion. This is supported by the observed findings showing that task performance was impaired for individuals lower in extraversion compared to those higher in extraversion under negative emotion conditions. These findings raise the possibility that individuals higher in extraversion may have protection against the detrimental influences of negative emotion.

In contrast to the hypotheses made, neuroticism did not impact change detection performance. Previous research has found that neuroticism is negatively correlated with attentional control (Bredemeier et al., 2011) and change detection accuracy (Hahn et al., 2015). However, in the current study, when the negative affect scores for individuals higher in neuroticism and lower in neuroticism were compared (for the negative emotion condition), no differences were evident between groups. This suggests that in the current study individuals higher in neuroticism and individuals lower in neuroticism were impacted to a similar degree by the negative emotion condition. This result complements the behavioral data showing that neuroticism had no impact on task performance. It may be that the influence of neuroticism on change detection is weaker than the influence of extraversion. For example, Hahn et al. (2015) showed that once eye movement measures were included in their regression model, a previously significant correlation between neuroticism and change detection was no longer significant.

Whilst the current work provides novel findings regarding the interactions between personality and emotion on visual attention, several limitations warrant consideration. We focused on the traits of extraversion and neuroticism as these traits are most closely associated with affective reactivity and models of psychopathology (American Psychiatric Association, 2013; Clark, 2005; Costa & McCrae, 1980; DeNeve & Cooper, 1998; Diener et al., 1992; Eysenck, 1990; John et al., 2008; Kokkonen & Pulkkinen, 2001; Watson et al., 1994). However, further research can investigate if trait levels of openness, agreeableness, and conscientiousness interact with emotion to influence visual attention. For example, higher trait levels of conscientiousness has been shown to be associated with improved accuracy during visual search (Biggs et al., 2017), yet it remains unknown whether conscientiousness could interact with emotion to guide the allocation of attention. Moreover, we hope that our initial work may stimulate additional research that can investigate the interaction between extraversion and emotion on visual attention in more detail. For instance, future research is required to test if specific facets of extraversion (e.g., warmth, gregariousness, assertiveness, activity, excitement seeking or positive emotion) are responsible for the interaction between emotion and extraversion observed in the current study. Lastly, whilst we conducted a sample size calculation to determine an appropriate sample size, the sample was modest. Future work should adopt larger sample sizes, particularly when investigating additional personality traits, or additional facets of extraversion or neuroticism.

To the best of the authors’ knowledge, the current study is the first to explore the effects of extraversion and neuroticism on the links between emotion and visual attention to account for some of the conflicting findings within the literature. Whilst some studies show that positive emotions expand visual attention, other work shows no impact of emotion on attention. It was proposed that because extraversion and neuroticism are associated with affective reactivity and emotional processing, they may moderate the effects of emotion on attention. This was tested using a change blindness flicker task in which participants searched for changes made to real‐world scenes under positive, negative, and neutral emotion states. The work replicated the basic findings of Bendall and Thompson (2015) showing no effect of emotion on attention in this task. Neuroticism had no overall impact on performance and did not interact with emotion. However, our novel findings show that higher trait extraversion was associated with increased performance (as reflected through higher accuracy). Negative emotion did impair performance, but this was only for individuals lower in extraversion, therefore there is some evidence that extraversion can moderate the influence of emotion on attention. The results suggest that the way in which emotion can influence attention may be partly determined by the ability of an individual to regulate their emotional state. This links to clinical findings reporting that those suffering from affective disorders show deficits in the allocation and control of attention.

### TRANSPARENT PEER REVIEW

The peer review history for this article is available at https://publons.com/publon/10.1002/brb3.2387

## CONFLICT OF INTEREST

The authors declare no conflict of interest.

## Supporting information

SUPPORTING INFORMATION

## Data Availability

The data that support the findings of this study are available from the corresponding author upon reasonable request.
