# Supplementary material for: Interactive influences of emotion and extraversion on visual attention
Source: Brain Behav. 2021 Oct 17;11(11):e2387. doi: 10.1002/brb3.2387 (PMC8613417; doi:10.1002/brb3.2387)
Supplement: Supplementary file 1 — SUPPORTING INFORMATION [file BRB3-11-e2387-s001.docx]

Interactive influences of emotion and extraversion on visual attention

*Bendall, R. C. A., Begley, S., and Thompson, C.

* Correspondence: Robert Bendall: [r.c.a.bendall@salford.ac.uk](mailto:r.c.a.bendall@salford.ac.uk)

**
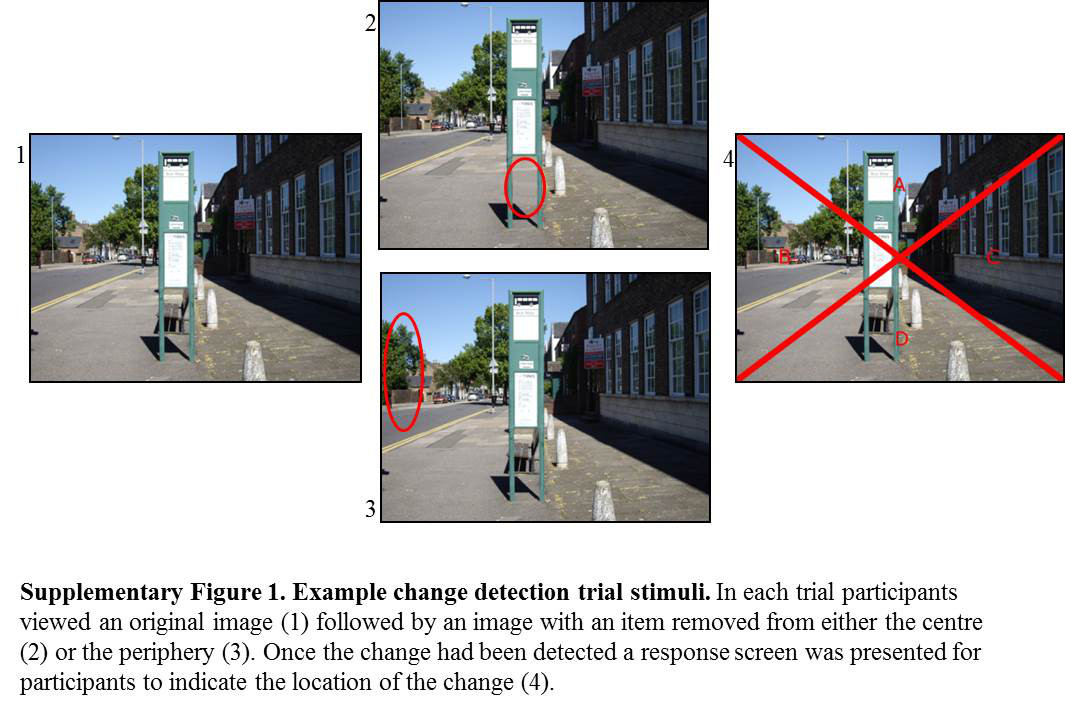
**

**Supplementary Table 1.** Emotional valence and arousal ratings of images used during emotion induction procedure taken from the International Affective Picture System (Lang et al., 2008).

| Positive images | | | Neutral images | | | Negative images | | |
| --- | --- | --- | --- | --- | --- | --- | --- | --- |
| IAPS number | Valence | Arousal | IAPS number | Valence | Arousal | IAPS number | Valence | Arousal |
| 1610 | 7.82 | 3.98 | 2101 | 4.49 | 3.46 | 2141 | 2.44 | 5.00 |
| 2058 | 7.91 | 5.09 | 2210 | 4.54 | 3.32 | 2205 | 1.95 | 4.53 |
| 2150 | 7.92 | 5.00 | 2215 | 4.63 | 3.38 | 2375 | 2.20 | 4.88 |
| 2314 | 7.55 | 4.00 | 2221 | 4.39 | 3.07 | 2900 | 2.56 | 4.61 |
| 2360 | 7.70 | 3.66 | 2383 | 4.72 | 3.41 | 3215 | 2.51 | 5.44 |
| 2398 | 7.48 | 4.74 | 5130 | 4.45 | 2.51 | 3220 | 2.49 | 5.52 |
| 2530 | 7.80 | 3.99 | 7025 | 4.63 | 2.71 | 9000 | 2.55 | 4.06 |
| 4622 | 7.46 | 4.11 | 7030 | 4.69 | 2.99 | 9007 | 2.49 | 5.03 |
| 5480 | 7.53 | 5.48 | 7031 | 4.52 | 2.03 | 9140 | 2.19 | 5.38 |
| 5700 | 7.61 | 5.68 | 7040 | 4.69 | 2.69 | 9332 | 2.25 | 5.34 |
| 5780 | 7.52 | 3.75 | 7044 | 4.69 | 3.94 | 9340 | 2.41 | 5.16 |
| 5829 | 7.65 | 4.68 | 7110 | 4.55 | 2.27 | 9419 | 2.55 | 5.19 |
| 7270 | 7.53 | 5.76 | 7130 | 4.77 | 3.35 | 9420 | 2.31 | 5.69 |
| 7502 | 7.75 | 5.91 | 7150 | 4.72 | 2.61 | 9421 | 2.21 | 5.04 |
| 7580 | 7.71 | 4.59 | 7180 | 4.73 | 3.43 | 9435 | 2.27 | 5.00 |
| 8200 | 7.54 | 6.35 | 7186 | 4.63 | 3.60 | 9560 | 2.12 | 5.50 |
| 8370 | 7.77 | 6.73 | 7224 | 4.45 | 2.81 | 9561 | 2.68 | 4.79 |
| 8380 | 7.56 | 5.74 | 7590 | 4.77 | 3.80 | 9810 | 2.09 | 6.62 |
| 8499 | 7.63 | 6.07 | 7705 | 4.77 | 2.65 | 9830 | 2.54 | 4.86 |
| 8502 | 7.51 | 5.78 | 8121 | 4.63 | 4.14 | 9901 | 2.27 | 5.70 |
|  | *M* = 7.65 | *M* = 5.05 |  | *M* = 4.62 | *M* = 3.11 |  | *M* = 2.35 | *M* = 5.17 |

**Supplementary Table 2.** Emotional valence and arousal ratings of positive images presented at the end of the experimental session taken from the International Affective Picture System (Lang et al., 2008).

| IAPS number | Valence | Arousal |
| --- | --- | --- |
| 1460 | 8.21 | 4.31 |
| 1710 | 8.34 | 5.41 |
| 1750 | 8.28 | 4.10 |
| 1920 | 7.90 | 4.27 |
| 2154 | 8.03 | 4.48 |
| 2332 | 7.64 | 4.30 |
| 2340 | 8.03 | 4.90 |
| 4626 | 7.60 | 5.78 |
| 5621 | 7.57 | 5.19 |
| 5760 | 8.05 | 3.22 |
| 5825 | 8.03 | 5.46 |
| 5830 | 8.00 | 4.92 |
| 5833 | 8.22 | 5.71 |
| 5910 | 7.80 | 5.59 |
| 7330 | 7.69 | 5.14 |
| 7405 | 7.38 | 6.28 |
| 8470 | 7.74 | 6.14 |
| 8496 | 7.58 | 5.79 |
| 8501 | 7.91 | 6.44 |
| 8540 | 7.48 | 5.16 |
|  | *M* = 7.87 | *M* = 5.13 |
